# Supra-Pubic Lithotomy

**Published:** 1877-09-20

**Authors:** C. W. Dulles


### SUPRA-PUBIC LITHOTOMY.

BY C. W. DULLES, M. D.

With report of a successful case, by E. F. Starr, M.D., of Nacoochee, Ga.

Nearly two years ago there appeared an article upon this subject in the *American Journal of the Medical Sciences* (July, 1875, p. 39), in which an attempt was made to set the merits of the operation in a somewhat better light than they have heretofore enjoyed. Following this, I have had the pleasure of receiving a number of letters, some narrating hitherto unreported cases, and others indicating that certain of the views then expressed met the approval of those who read them. Among these letters was one from Dr. E. F. Starr, of Nacoochee, Ga., who wrote that he had a patient upon whom he must operate, asked for further elucidation of some of the statements in the article alluded to, and subsequently performed the supra-pubic operation with perfect success, and sent me a full report of his case.

The report, with his consent, I now present, almost in his own words, and wish to take the opportunity to add a few remarks to what has been already published in regard to the method of operating.

Case. Mr. ——, æt. 35, a large athletic man, had been an invalid for the last two or three years, but thought he had felt symptoms of stone for twelve years; for two or three years had been subject to occasional attacks of cystitis, setting in with shivering and resulting in pyrexia and difficult micturition, which caused him a great deal of suffering; under judicious treatment, he had for the last two or three months escaped these paroxysms and improved in general health, so that at the time of the operation he weighed two hundred pounds.

On the 6th of May, 1876, with the patient under the influence of chloroform, I proceeded to perform supra-pubic lithotomy. The patient was laid upon his back on a low couch, with his feet hanging down and resting on the lower round of a chair, while I sat by his right side and made the first incision from above downward. As I expected the abdominal parietes to be thick, I made my cut about four inches in length.

Some small arterial branches spouted a little blood, which was carefully sponged away, and, before entering the pelvic cavity further time was given for the general oozing to cease. After this, using the finger as a guide, and carefully slitting the tissues as I proceeded, the bladder wall was reached, the peritoneum being entirely avoided.

The feet were then elevated to the top of the couch, a sound introduced through the urethra, and the bladder raised up between the lips of the incision. The amount of fatty tissue enveloping the viscus made it somewhat difficult to denude; but, as the peritoneum had been avoided, this was not deemed indispensable, and when fairly cleared it was seized with a tenaculum, punctured just below with a sharp-pointed bistoury, and then incised to a sufficient extent with a Blizard's knife upon a grooved director.

The bladder had been ordered to be emptied before the administration of the anæsthetic, but the kidneys being in a very active state from the previous free use of diruetic and demulcent drinks, and the time required to produce anæsthesia having been considerable, in addition to that consumed in making the incisions, some urine was found to have accumulated, and a part of it escaped through the external wound. But as this was pretty well filled by the bladder as it was held up, the urine passed freely out.

The finger was then introduced and the stone brought out upon its palmar surface. It was smooth, of a flat oval shape, and weighed one ounce and one drachm.

The thickness of the abdominal walls made the wound appear formidable, and its closure promise difficulty. This, however, was not so great as was anticipated.

At this point of the operation I departed from the plan proposed in the article on Supra-pubic Lithotomy, in the *Am. Journal of Med. Sciences* for July, 1875; and instead of closing the bladder alone by the Lembert suture, I *closed both the bladder and the parietes of the abdomen at once* by that suture. That is to say, I passed a silver suture down through the wall of the abdomen into the cavity of the bladder, included a part of this and brought the wire back through the bladder and abdominal wall on the same side; then I carried it across the incision, passed it down through the abdominal wall and bladder on this side, included a segment here, and brought it out as before and just opposite where it had first entered the tissues. Now, when the ends of the suture were drawn upon, the sides of the wound were approximated, but the edges of the incision in the bladder were inverted and their outer surfaces brought into contact while the mucous surfaces were turned inward, thus promoting union.

The effect of this suture in common is to prevent infiltration of urine by keeping the bladder in close contact with the abdominal wall, while it also tends to secure general union by first intention and thereby speedy recovery.

Of course one case is not sufficient to establish a rule, but the practical working of this one tended to confirm me in my belief that the supra-pubic operation, performed in this way, with the necessary after care, may be more speedily recovered from, with less attending danger, than the perineal. I am anxious for an opportunity to put this new feature to a thorough test. I may mention, in this connection, that I prefer to have the bladder empty, and raise it with the sound, so that when the incision is made in it the urine shall not flow out into the wound, though it would do little harm if it does not infiltrate, or get between the bladder and abdominal wall. In another case I should make the first incision from below upwards, because in this way the regional anatomy can be best observed and appreciated, and the cavity more readily entered near the pubes and the peritoneum avoided.

An hour after the conclusion of the operation, the patient being in bed, I introduced an English catheter into the bladder, as I thought, but no urine flowed. Leaving him for a while, I was sent for in an hour or two with the announcement that he wished to pass water. I introduced the flexible catheter again, but no urine came, and when I withdrew the instrument he remarked that he thought he could pass it himself, and allowed it to flow, lying upon his side. While he was doing this there seemed to be a spasmodic action of the parts concerned in its expulsion, and there was a gush of urine from the wound. Being already sick from the effects of the chloroform, he now became faint, and looked quite ghastly for a little while. So I removed the strips of plaster and a superficial suture from the lower end of the wound to allow a free exit. I then instituted drainage by filling a small French catheter with water, passing it through the urethra into the bladder, and turning its end down into a cup placed between his legs. This was late in the afternoon. He had a somewhat restless night, and on the following day some fever, which was easily controlled with veratrum.

Late in the afternoon of the second day, being apprehensive of burrowing of urine between the bladder and pubes —although there was no local symptoms of it—I was induced to remove another suture—the lower deep one. Down to this point adhesion was taking place very nicely, and, had all the sutures been allowed to remain, I think the whole wound would have united as favourably as could have been wished. As it was, there was some gaping at the lower end from the severing of the adhesions that had taken place, which retarded recovery. At the expiration of ten days the catheter was finally removed, and he got on his feet and passed his urine per urethram. At this time all the remaining sutures were removed except one, which was left in until the sixteenth day, when it was taken away, and the patient discharged cured.

During the whole time there was no inflammation, nor swelling, nor indication of burrowing, or abscess about the wound, and it seems to me clear that this method of closing the wound is far preferable, both in point of time and safety, to that of leaving either one or both wounds open.—*Amer. Jour. Med. Sciences.*

